# An Integrated Approach Based on Network Analysis Combined With Experimental Verification Reveals PI3K/Akt/Nrf2 Signaling Is an Important Way for the Anti-Myocardial Ischemia Activity of *Yi-Qi-Tong-Luo* Capsule

**DOI:** 10.3389/fphar.2022.794528

**Published:** 2022-02-16

**Authors:** Huxinyue Duan, Meiyan Li, Jia Liu, Jiayi Sun, Chunjie Wu, Yu Chen, Xiaohui Guo, Xinglong Liu

**Affiliations:** ^1^ Chengdu University of Traditional Chinese Medicine, Chengdu, China; ^2^ Guangyuan Hospital of Traditional Chinese Medicine, Guangyuan, China; ^3^ Hospital of Chengdu University of Traditional Chinese Medicine, Chengdu, China

**Keywords:** apoptosis, myocardial ischemia, network analysis, oxidative stress, Yi-Qi-Tong-Luo Capsule

## Abstract

**Background:** *Yiqi-Tongluo Capsule* (YTC) is a Chinese traditional patent medicine that has been used in the treatment of myocardial ischemia (MI). However, its molecular mechanisms against MI have not been clear.

**Methods:** Network analysis and experimental verification were used to explore the potential molecular mechanisms of YTC for MI treatment. Firstly, the main components in the capsules and the potential targets of these components were predicted by online databases. The MI related genes were collected from Genecards and Online Mendelian Inheritance in Man (OMIM) databases. The drug targets and disease targets were intersected, and then the protein-protein interaction (PPI) and Drug-Molecular-Target-Disease Network (DMTD) were constructed, and GO enrichment analysis and KEGG pathway enrichment analysis were performed. Based on the H_2_O_2_-stimulated H9c2 cells, flow cytometry, western blot (WB) and immunofluorescence experiments were performed to verify the network analysis prediction.

**Results:** A total of 100 active components and 165 targets of YTC were predicted, in which there were 109 targets intersected with the targets of MI. GO and KEGG analysis showed that these potential targets were related to a variety of biological processes and molecular mechanisms, including oxidative stress and PI3K/AKT pathway. Astragaloside IV (AS IV) and paeoniflorin (PAE) might be the main active components in YTC. The results of cell counting kit-8 (CCK-8) showed that YTC alleviated the damage of H_2_O_2_ to H9c2 cells. The results of flow cytometry, DAPI staining and JC-1 probe showed that YTC alleviated H_2_O_2_ induced apoptosis in H9c2 cells. In addition, YTC reduced the level of intracellular superoxide anion, increased the activities of superoxide dismutase (SOD), catalase (CAT) and glutathione peroxidase (GSH-Px), and reduced the content of malondialdehyde (MDA) in H_2_O_2_-induced H9c2 cells. The results of immunofluorescence and WB showed that the phosphorylation of PI3K and Akt were increased, the expression of Bcl-2 was up-regulated and the expression of cleaved caspase-3 and Bax were down-regulated. Besides, the nuclear translocation of Nrf2 were increased.

**Conclusion:** In conclusion, the results of this study showed that YTC might alleviate MI by suppressing apoptosis induced by oxidative stress via the PI3K/Akt/Nrf2 signal pathway.

**Graphical Abstract:**
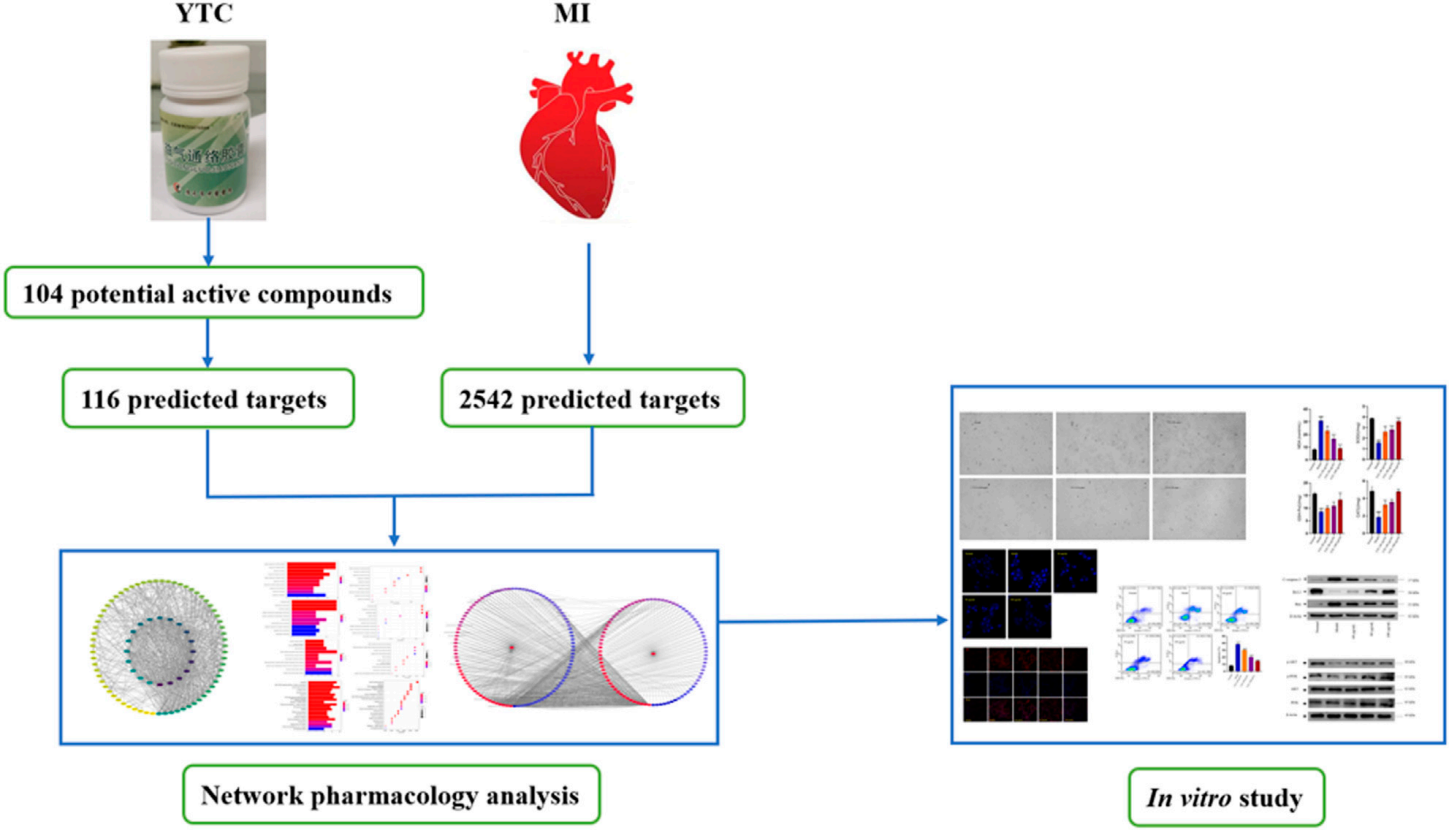


## Introduction

Myocardial ischemia (MI) is a physical condition in which the blood perfusion of heart is decreased, resulting in insufficient blood flow, insufficient oxygen supply and abnormal energy metabolism (Fan et al., 2019). As a common cardiovascular disease, MI can lead to irreversible myocardial damage, which has high incidence rate and mortality rate worldwide (Li et al., 2021). It has been reported that the pathogenesis of MI involves a variety of biological processes, including oxidative stress, apoptosis, inflammatory and immune responses (Zhao and Vinten, 2002; Kaul and Ito, 2004; Arslan et al., 2011; Yang et al., 2014). Currently, nitrates, calcium antagonists and β-blockers are the drugs commonly used in cardiovascular diseases; nonetheless, the mortality rate caused by MI is still high all over the world (Malakar et al., 2019). Therefore, it is necessary to identify more safe and effective drugs for MI. With a long history of clinic use, Traditional Chinese medicine (TCM) has great advantages to treat cardiovascular diseases (Duan et al., 2021; Zhang et al., 2021a), which can be seen as a resource library for alternative drugs of MI (Wu et al., 2020).

*Yiqi-Tongluo* Capsule (YTC) is a Chinese patent medicine of the Guangyuan Hospital of traditional Chinese medicine, and it has been used for decades in the treatment of MI with reliable therapeutical effects (Zhang, 2015). YTC is consisted of the roots of *Astragalus membranaceus* (Fisch.) Bge. var. mongholicus (Bge.) Hsiao (also called *Huangqi* in Chinese), the roots of *Angelica sinensis* (Oliv.) Diels (also called *Danggui* in Chinese), the body of *Pheretima aspergillum* (E. Perrier) (also called *Dilong* in Chinese), the root of *Paeonia lactiflora* Pall. (also called *Chishao* in Chinese), the root of *Codonopsis pilosula* (Franch.) Nannf. (also called *Dangshen* in Chinese), the roots of *Ligusticum chuanxiong* Hort. (also called *Chuanxiong* in Chinese), the flower of *Carthamus tinctorius* L. (also called *Honghua* in Chinese), et al. It has been reported that extracts from *A. membranaceus* improved cardiac function in a rat model of MI by reducing myocardial cells apoptosis (Ma et al., 2013). Another study showed that astragalus polysaccharides alleviated inflammation and necrosis of myocardium in myocardial ischemia-reperfusion rats (He et al., 2018). In addition, *A. sinensis* polysaccharides were reported had cardioprotective activity in myocardial ischemia-reperfusion rats via its capacity of antioxidant, up-regulation of CT-1 and down-regulation of caspase-12 mRNA (Zhang et al., 2010). However, the active ingredients and molecular mechanisms of YTC are still unclear, which limits its further application and promotion.

Network analysis is an emerging technology, which integrates a variety of disciplines, including system biology, multi-directional pharmacology, network analysis and computational biology (Hopkins, 2008). In this approach, based on the interaction between drugs, components, targets and disease, incorporates biological networks and drug action networks are established to analyze and predict the potential active ingredients and mechanism of action of drugs (Xu et al., 2014). TCM is characterized by multi-components and multi-targets, which cause difficulty in illustrating the specific mechanism of TCM. Therefore, it is suitable to use network analysis to help us systematically study TCM and provide scientific basis for the research of TCM. In this study, the main components of YTC, the potential targets and underlying mechanisms were predicted by network analysis. And the predicted results of network analysis were verified by vitro experiments. The above study not only reveal the potential chemical and pharmacological basis of YTC, but also provides reference for the development and promotion of Chinese patent medicine. The main process of this study is presented in Graphical Abstract.

## Materials and Methods

### Chemicals, Reagents, and Materials

All herbal medicines were provided by Guangyuan Hospital of Traditional Chinese Medicine (Guangyuan, China). Fetal bovine serum (FBS) and Dulbecco’s modified Eagle medium (DMEM) were purchased from the Gibco Co. (Grand Island, NY, United States). BCA protein assay reagents, SDS-polyacrylamide gel electrophoresis (SDS-PAGE) preparation kit, penicillin-streptomycin mixture, trypsin, phosphate buffer saline (PBS) and cell counting kit-8 (CCK-8) were purchased from Boster Biological Technology Co., Ltd. (Wuhan, China). Annexin V-FITC/PI assay kit was purchased from Multisciences (Lianke) Biotechnology Corporate Limited (Hangzhou, China). H_2_O_2_ was purchased from Chengdu Chron Chemicals Co. Ltd. (Chengdu, China). Primary antibodies for PI3K, phosphorylation-PI3K (p-PI3K), AKT, phosphorylation-AKT (p-AKT), Nrf2, Bcl-2, Bax and cleaved-caspase-3 (C- caspase-3) were obtained from the ImmunoWay Biotechnology Co. (Suzhou, China). The assay kits for MDA, SOD, CAT, and GSH-PX were purchased from the Nanjing Jiancheng Bioengineering Institute (Nanjing, China). Ultrapure water purified by Millipore Ultra-pure Water Purifier (Millipore, Milford, MA, United States) was used. Other reagents were all of analytical grade.

### Preparation of *Yiqi-Tongluo* Capsule Freeze-Dried Powder

All the dried herbal medicines including the roots of *Astragalus membranaceus* (Fisch.) Bge. var. mongholicus (Bge.) Hsiao (304 g), the roots of *Angelica sinensis* (Oliv.) Diels (190 g), the body of *Pheretima aspergillum* (E. Perrier) (114 g), the root of *Paeonia lactiflora* Pall. (152 g), the root of *Codonopsis pilosula* (Franch.) Nannf. (304 g), the roots of *Ligusticum chuanxiong* Hort. (190 g), the flower of *Carthamus tinctorius* L. (76 g), and the aboveground parts of *Mentha haplocalyx* Briq (76 g), were powdered and extracted with water by decoction for 3 times (1 hour each time). Then, the decoction was filtered and concentrated under 60°C to afford the thick paste with a vacuum rotary evaporator (EYELA, N-1300V, Tokyo, Japan). Subsequently, a lyophilizer (Labconco Co., Kansas, MI, United States) was used to freeze-dry the extracts to obtained the powder, which was sealed and stored at 4°C (yield 30%) for subsequent experiments.

### Network Analysis

#### Screening of *Yiqi-Tongluo* Capsule Active Ingredients and Targets

The potential active components of YTC were screened from the Traditional Chinese Medicine Integrated Database (TCMSP, https://old.tcmsp-e.com/tcmsp.php). Subsequently, the ChemDraw professional 15.0 were used to draw the chemical structures. Then, the Mol. format files were uploaded to SwissTargetPrediction (http://www.swisstargetprediction.ch/) and TCMSP (https://old.tcmsp-e.com/tcmsp.php) online databases to obtain potential targets. In addition, some important components and targets of single drugs in YTC reported in literatures were supplemented (Sun et al., 2012; Qu et al., 2016; Bai et al., 2020; Wang et al., 2020).

#### To Establish Myocardial Ischemia Targets Library

The key word “myocardial ischemia” was input into the Online Mendelian Inheritance in Man database (OMIM) and the Genecards database (http://www.genecards.org) to screen target genes of MI. Then, the collected target genes were converted into standard gene names through the UniProt database (http://www.uniprot.org/).

#### Protein-Protein Interaction

Then, the potential targets of components in YTC and MI-related genes were overlapped as the objective targets. The PPI analysis was performed by the online STRING database (https://string-db.org/). The results of PPI analysis were updated into Cytoscape software (ver.3.7.1) to visualize these interactions and construct PPI network. Furthermore, CytohHubba plug-in tool was used to analyze the important proteins in PPI.

#### Drug-Molecular-Target-Disease Network Construction

Selected compounds and targets were uploaded into Cytoscape software (ver.3.7.1) to construct the Drug-Molecular-Target-Disease Network (DMTD) diagram. In this network diagram, nodes shoed the drug, disease, ingredients and genes, while edges represented the relationships between them. Finally, the importance of each node in the network was evaluated by calculating the degree, average shortest path length and closeness centrality in DMTD.

#### Bioinformatic Analysis

The clusterprofiler software package was downloaded in R language software to analyze the gene ontology (GO) term function and the Kyoto Encyclopedia of genes and genomes (KEGG) pathway enrichment, so as to obtain the function and pathway of the collected proteins. In this study, the criterion of statistically significant was *p* < 0.05, and only the top 10 were shown in the results.

### *In vitro* Experiment

#### Cell Culture and Treatment

The H9c2 cells, a kind of cardiac myoblast isolated from rat, were purchased from Shanghai Yubo Biotech Co., Ltd. (Shanghai, China), and cultured in DMEM with FBS, 100 U/mL penicillin and 100 μg/ml streptomycin. Cells were cultured in a 37°C incubator with 95% air and 5% CO_2_, and were subcultured every 2–3 days. Only cells in the exponential growth stage were used in this study.

Different concentrations of YTC (60, 80, and 100 μg/ml) were used to pretreat H9c2 cells for 24 h and then 200 μM H_2_O_2_ (Zhang et al., 2018) was incubated with cells for another 4 h. In the model group, YTC was replaced by the same amount of DMEM medium.

#### Cell Viability Assay

Cell viability was measured by a CCK-8 kit. H9c2 cells were seeded in a 96 well plate at 1×10^4^ cells/well, and then treated with different concentrations of YTC (40, 60, 80, 100 and 120 μg/ml) for 24 h. After removing the medium containing YTC, 10 μl CCK8 solution and 90 μl fresh medium were added, and cells were incubated for 30 min in the incubator. At last, the optical density (OD) values were determined by a microplate reader at 450 nm. After that, H9c2 cells were pretreated with different concentrations of YTC (40, 60, 80, 100 and 120 μg/ml) for 24 h, and then treated with 200 μM H_2_O_2_ for 4 h.

#### Nuclear Staining with DAPI

H9c2 cells were incubated in a 6-well plate (1×10^5^ cells/well) and pretreated with YTC (60, 80 and 100 μg/ml) for 24 h, and subsequently treated with 200 μM H_2_O_2_ for 4 h. After that, the cells were washed twice with PBS, and then fixed with 4% polyoxymethylene for 15 min. After fixation, the cells were incubated with DAPI solution in dark room for 10 min. Then, cells were washed with PBS and observed under a fluorescence microscope.

#### Apoptosis Assay by Flow Cytometer

The cells were incubated in a 6-well plate (1×10^5^/well) and then treated as described above. Then, the cells were collected and washed with PBS, and the supernatant was removed by centrifugation. The diluted Annexin V binding buffer working solution was used to resuspend cells, and then 2.5 μl Annexin V-FITC and 2.5 μl PI staining solution was added. After mixed well and incubated at room temperature in the dark for 20 min, the apoptosis rate of cells was detected by a flow cytometry (Beckman Coulter Company, Brea, CA, United States).

#### Influence of Main Active Components on H9c2 Cells

According to the results of DMTD and many reported articles, two potential active components were selected as the main components of YTC, including astragaloside IV (AS IV) and paeoniflorin (PAE). Firstly, the effects of these two compounds on the viability of H_2_O_2_-induced H9c2 cells were detected by a CCK-8 kit. Then the anti-apoptosis effects of these components were detected by a flow cytometry. The experimental steps of CCK-8 and flow cytometry were described above.

#### Determination of Mitochondrial Membrane Potential

The cells were seeded in laser confocal dishes (1×10^4^/well) and then treated with YTC and H_2_O_2_ as described above. Then, 10 μg/ml JC-1 probe was added and incubated for 30 min under dark conditions. Subsequently, after washing twice with PBS, cells were observed under a laser confocal microscope (Leica, SP8 SR, Wetzlar, Germany) to obtain the fluorescence images.

#### Detection of Intracellular ROS

The fluorescence probe dihydrogen ingot (DHE) was used to detect intracellular superoxide anion levels. Briefly, H9c2 cells were seeded in laser confocal dishes (1×10^4^/well) and treated with YTC and H_2_O_2_ as described above. Subsequently, cells were incubated with 10 μM DHE for 20 min, and subsequently cultured with DAPI solution for 10 min. Then, the cells were washed with PBS and observed by a laser confocal microscope (Leica, SP8 SR, Wetzlar, Germany).

#### Determination of MDA, SOD, GSH-Px and CAT

Commercial assay kits were used to assay these antioxidant indexes according to the instructions. In brief, cells were seeded in 6-well plates (1×10^5^ cells/well) and treated with H_2_O_2_ and YTC as described above after incubated for 24 h. Then the RIPA lysis buffer was added to lyse cells, and the supernatant was collected after centrifuging to obtain the total protein. Subsequently, the level of MDA and activities of SOD, GSH-Px and CAT were assayed by assay kits according to the steps described in instructions.

#### Detection of Nuclear Translocation of Nrf2

The H9c2 cells were seeded in laser confocal plates (1×10^4^/well) and treated with YTC (60, 80 and 100 μg/ml) and H_2_O_2_ (200 μM) as described above. Then, the cells were incubated with 4% paraformaldehyde for 30 min and subsequently infiltrated with 0.3% Triton for 30 min. After that, cells were incubated with 10% serum for 1 h. Then, the samples were incubated overnight with primary antibody Nrf2 at 4°C. Subsequently, the samples were washed with PBS for 3 times and incubated with fluorescent secondary antibody for 2 h. Finally, DAPI sealing agent was added and the samples were imaged under a laser confocal microscope (Leica, SP8 SR, Wetzlar, Germany).

#### Western Blotting Assay

After treating as described above, cells were collected and proteins were obtained by adding 100 μl RIPA lysis buffer at the end of the treatment. A BCA protein assay kit was used to assay the protein concentrations of the supernatants. Then, SDS-PAGE was prepared to separate the protein, and the separated protein was transferred onto polyvinylidene fluoride (PVDF) membrane. Then, the PVDF membrane was blocked with 5% fat-free milk at room temperature, and was subsequently incubated with primary antibodies of Bcl-2 (dilution of 1:1,000), Bax (dilution of 1:1,000), C-caspase-3 (dilution of 1:1,000), Akt (dilution of 1:1,000), p-Akt (dilution of 1:1,000), PI3K(dilution of 1:1,000) and p-PI3K(dilution of 1:1,000). After washed for three times, the membrane was incubated with HPR-conjugated antibody (1:5,000) for 1 hour. Finally, the membrane were imaged under an enhanced chemiluminescence (ECL) system, and β-actin was used as the internal reference. Gray analysis of each blot was performed by the ImageJ software (version 1.51, National Institutes of Health, MD, United States).

#### Immunofluorescence Assay

The H9c2 cells were seeded in laser confocal plates (1×10^4^/well) and treated with YTC (60, 80 and 100 μg/ml) and H_2_O_2_ (200 μM) as described above. Then, the cells were incubated with 4% paraformaldehyde for 30 min and subsequently washed with PBS for 3 times. Then, the H9c2 cells were infiltrated with 0.3% Triton for 30 min. After that, cells were incubated with 10% serum for 1 h. Then, the samples were incubated overnight with primary antibody PI3K, p-PI3K, Akt, p-Akt, Bcl-2 and Bax at 4°C. Subsequently, the samples were washed with PBS for 3 times and incubated with fluorescent secondary antibody for 2 h. Finally, DAPI sealing agent was added and the samples were imaged under a laser confocal microscope (Leica, SP8 SR, Wetzlar, Germany).

#### Statistical Analysis

The results of this study were expressed as mean ± SD. Student’s t-test and one-way analysis of variance (ANOVA) were used to analyze these data, and *p* < 0.05 was significant. Each experiment was repeated three times.

## Results

### Chemical Composition of YTC Extract

161 components were collected, including 19 components in Huangqi, 3 components in Danggui, 3 components in Dilong, 22 components in Chishao, 18 components in Dangshen, 26 components in Chuanxiong, 15 components in Honghua, 4 components in Taoren, 33 components in Jiangxiang, 7 components in Xiebai, 1 components in Gualoupi and 10 components in Bohe. After the repetitive components were eliminated, 146 components were obtained (Supplementary Table S1).

### Target Prediction of YTC Against MI

**FIGURE 1:**
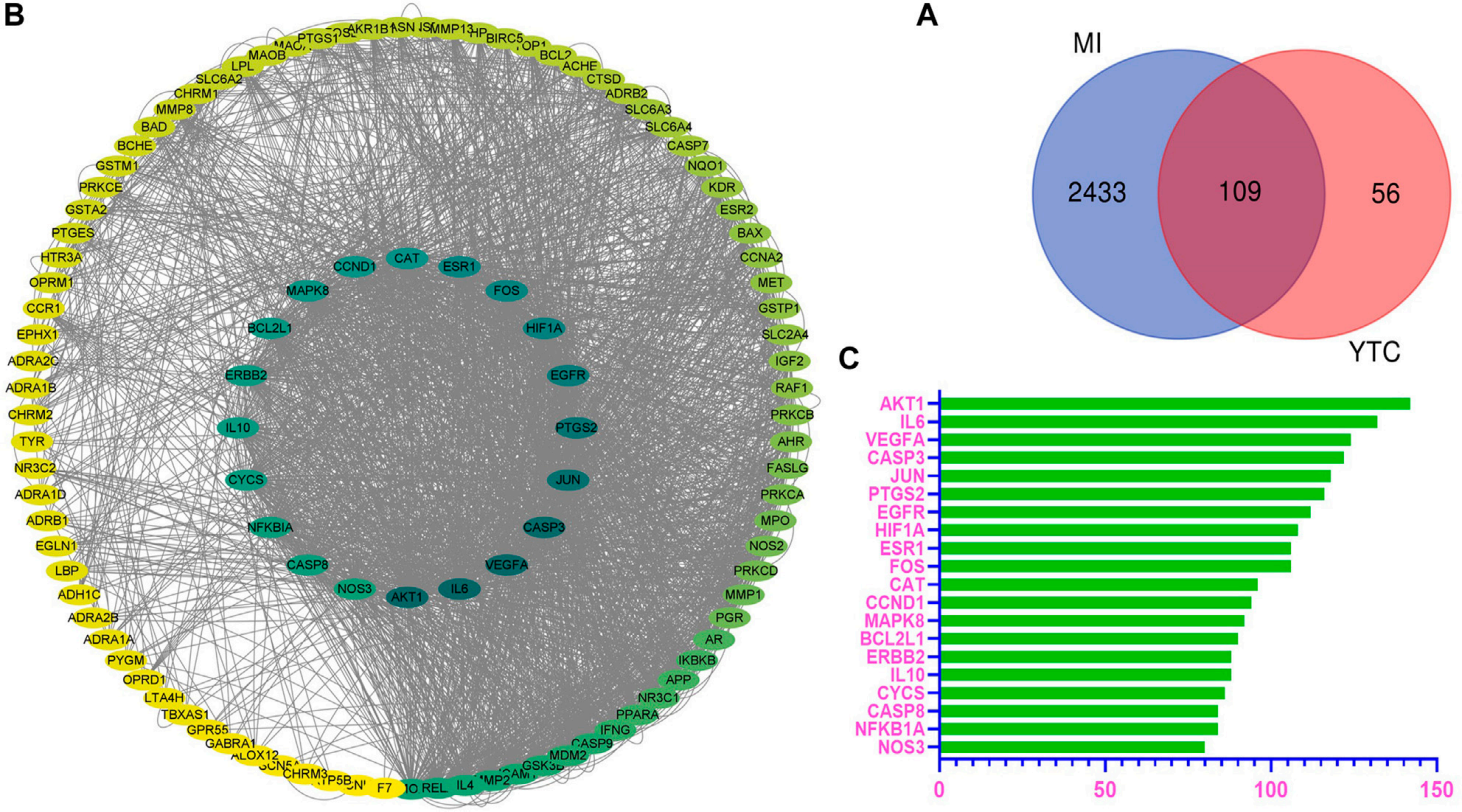
Overlapped targets screen. **(A)** The protein-protein interaction (PPI) network of targets. The colors of the nodes are illustrated from green to yellow in descending order of degree values. **(B)** Overlap targets of YTC and MI. The blue circles represent MI targets and the red circles represent YTC targets. The overlapped area is the targets of YTC anti-MI. **(C)** The top 20 genes in the PPI network were selected based on the degree.

From online databases and previous reports, 165 targets of YTC were collected after deleting duplicated targets. And a total of 2542 MI-associated genes were collected from the OMIM and Genecards database. Subsequently, combining the potential target genes of YTC with MI-associated target genes, 109 overlapped genes were gained, as shown in the Venn diagram (Figure 1A). The 109 genes were considered the molecular targets of YTC against MI.

### Results of PPI Network Analysis

Based on the potential protein targets of YTC against MI, the PPI network was constructed by STRING database and was entered into Cytoscape (ver.3.7.1) for visualization. As shown in Figure 1B, the edges represent the interaction between the two proteins, and the node represents the target proteins.

The most important 20 targets were selected according to node degree, as shown in Figure 1C, including AKT1, IL6, VEGFA, CASP3, JUN, PTGS2, EGFR, HIF1A, ESR1, FOS, CAT, CCND1, MAPK8, BCL2L1, ERBB2, IL10, CYCS, CASP8, NFKBIA, NOS3, indicating these genes might be the main potential targets of YTC.

### GO and KEGG Enrichment Analyses

**FIGURE 2:**
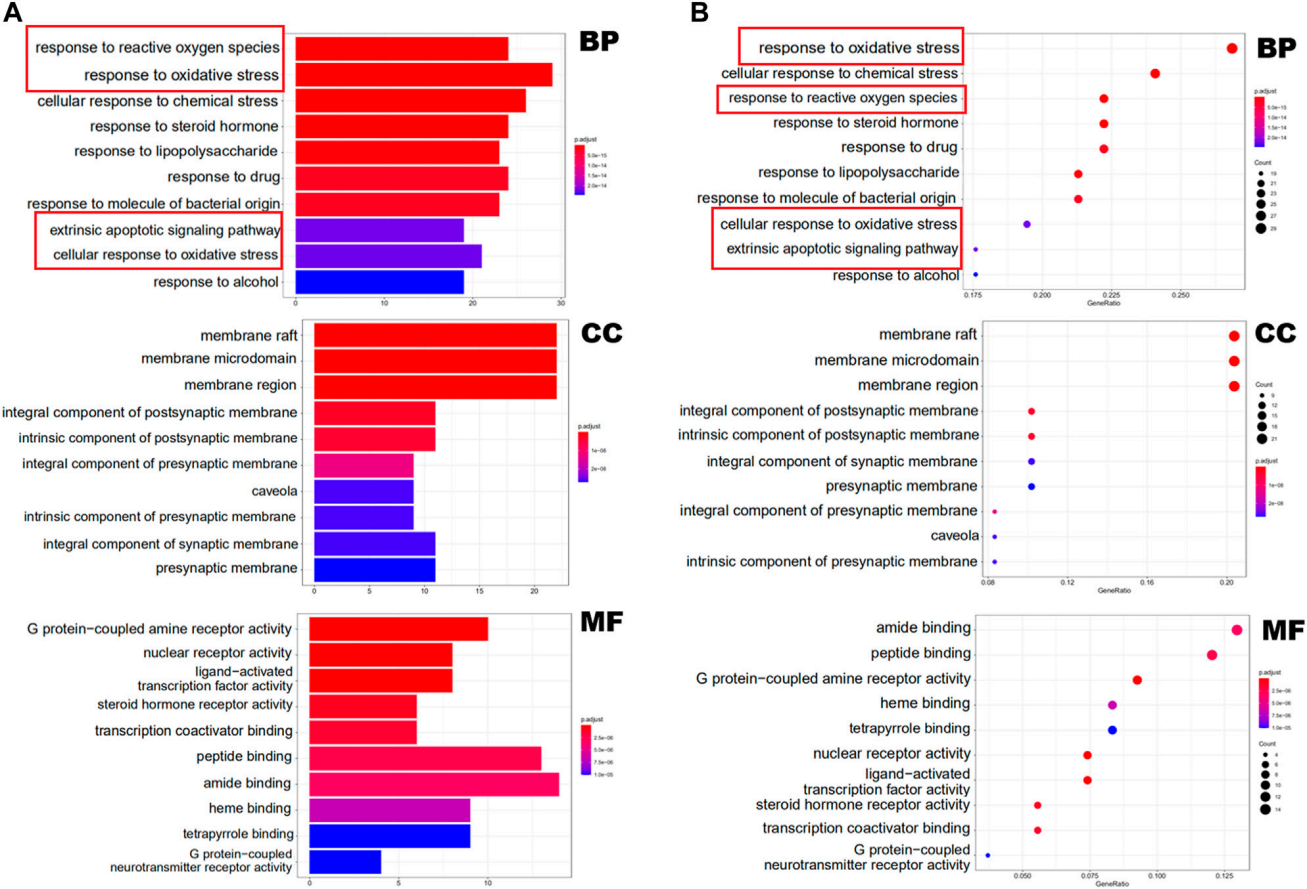
GO functional enrichment analysis represented in bar chart **(A)** and bubble diagram**(B)**.

In Figure 2, the terms of GO are located on the *y*-axis, and the number on the *x*-axis represents the degree of enrichment. The color of red indicates high reliability, whereas the color of blue indicates low reliability. The results of GO analysis indicates that the biological processes (BPs) of these targets are mainly in the response to reactive oxygen species, oxidative stress and chemical stress. Cellular components (CCs) are mainly related to membrane raft, membrane microdomain and membrane region. The main molecular functions (MFs) are the G protein-coupled amine receptor activity, nuclear receptor activity and ligand-activated transcription factor activity (Supplementary Table S2).

**FIGURE 3:**
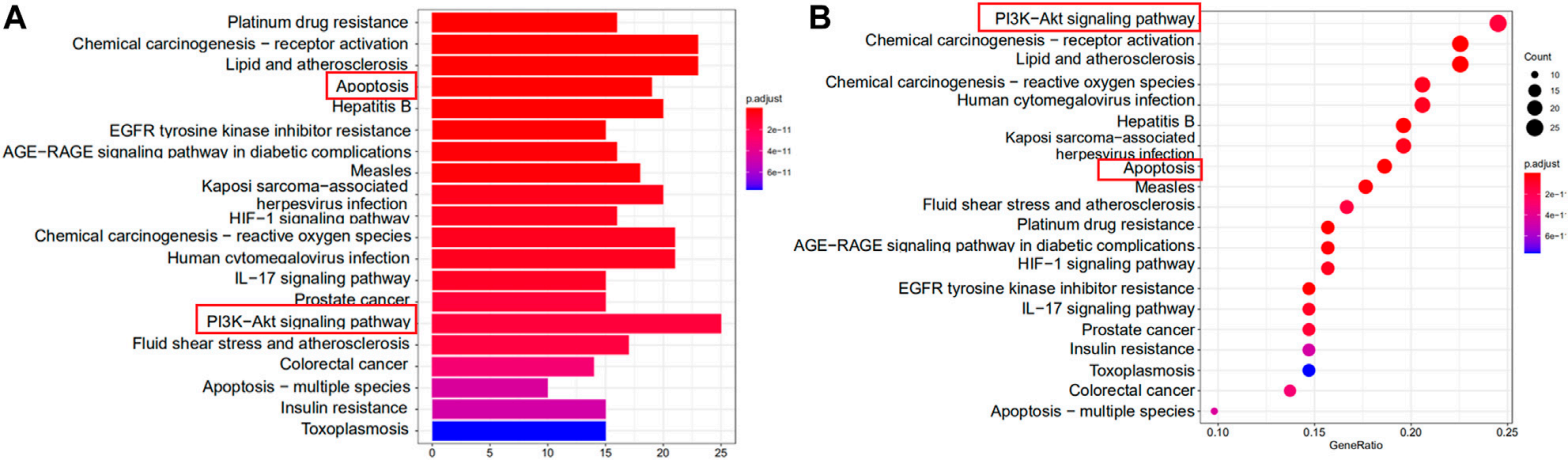
KEGG functional enrichment analysis represented in bar chart **(A)** and bubble diagram **(B)**.

Enrichment analysis of KEGG pathway of the 109 potential therapeutic targets was performed to further identify the potential pathways, and the detail information was presented in Supplementary Table S3. The most important 20 signaling pathways are shown in Figure 3, including platinum drug resistance, chemical carcinogenesis-receptor activation, lipid and atherosclerosis, apoptosis, hepatitis B, measles, prostate cancer and PI3K-Akt signaling pathway. Among them, apoptosis signaling pathway and PI3K-Akt signaling pathway were mainly found to be involved in apoptosis or oxidative stress biological processes related to MI (Zhai et al., 2017; Yuan et al., 2020). In addition, the analysis results of GO also suggested that oxidative stress might be an important biological process of YTC to against MI. Combined with PPI analysis and previous literatures, it was shown that CASP3, MAPK8, FOS, HSP90AA1, NOS3, ERBB2 and CASP8 were associated with apoptosis or oxidative stress in MI (Sato et al., 2001; Scarabelli et al., 2002; Wei et al., 2011; Zhu et al., 2016; Bai et al., 2019; Ma and Jin, 2019). Therefore, we established a H_2_O_2_ stimulated H9c2 cell model *in vitro* to further explore and study the myocardial protection effects of YTC.

### The Main Potential Active Component Against MI in YTC

**FIGURE 4:**
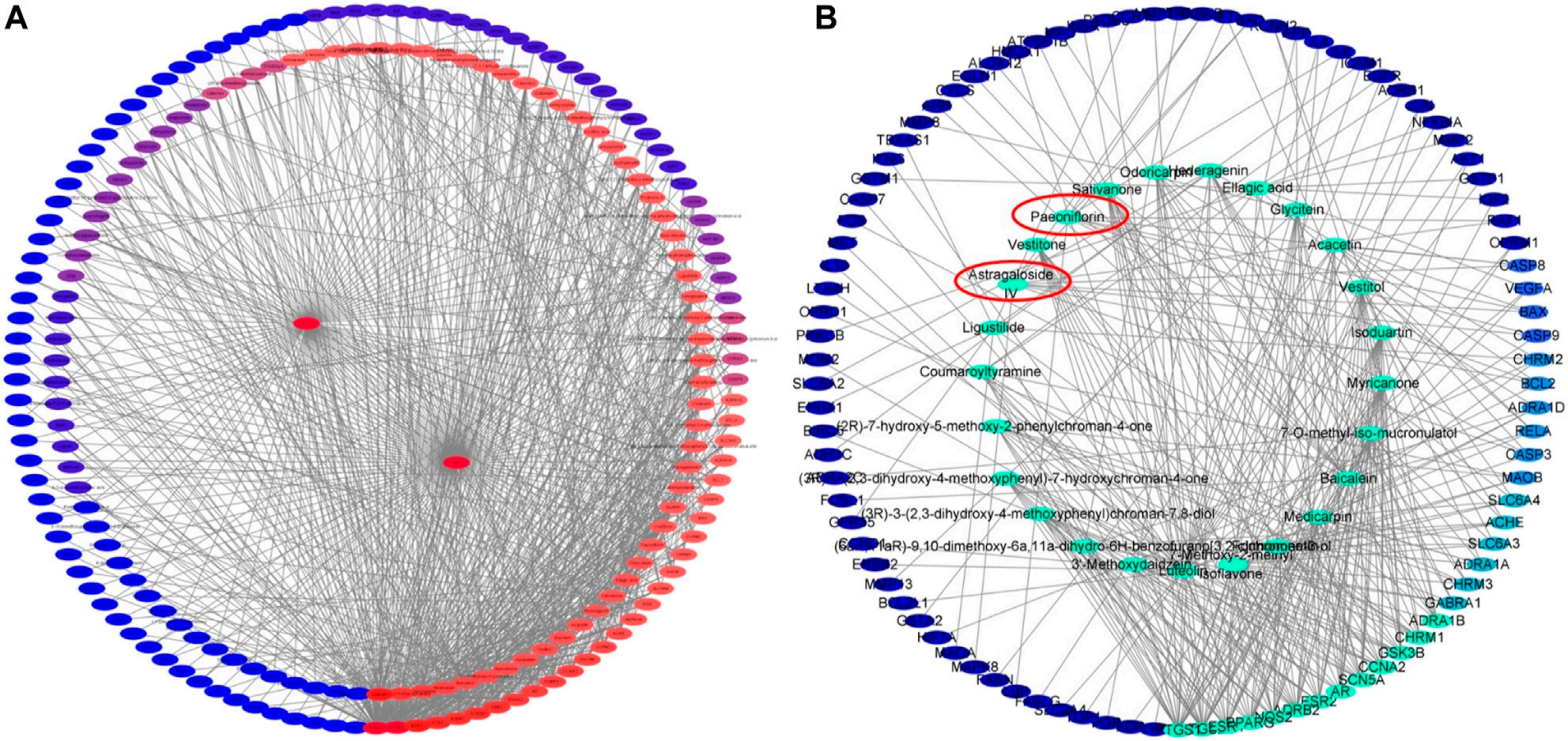
Network analysis of potential active compounds and targets. **(A)**The drug, active compounds, disease and targets were input into Cytoscape to construct the DMTD diagram, and the colors of the nodes are illustrated from red to blue in descending order of degree values. **(B)** The top 25 components and their related targets in the DMTD network were selected based on the degree, and the colors of the nodes are illustrated from green to blue in descending order of degree values.

As shown in Figure 4A, the DMTD diagram contained 193 nodes and 817 edges, including 1 disease, 1 drug, 100 components and 109 targets. The right circle consisted of targets, while the left circle consisted of components. The color changed from blue to red meant a greater number of connections and more important the connection is. The 100 components of YTC acted on different targets at the same time, which indicated that the effect of YTC on MI had the characteristics of multi-components and multi-targets. In addition, the top 25 components and related targets were selected, as shown in Figure 4B **.** According to the DMTD diagram and previous reports, AS IV and PAE, the characteristic components in YTC, were selected as the potential main active components and verified in subsequent experiments.

### Experimental Validation In Vitro

#### Effect of YTC on Cell Viability

**FIGURE 5:**
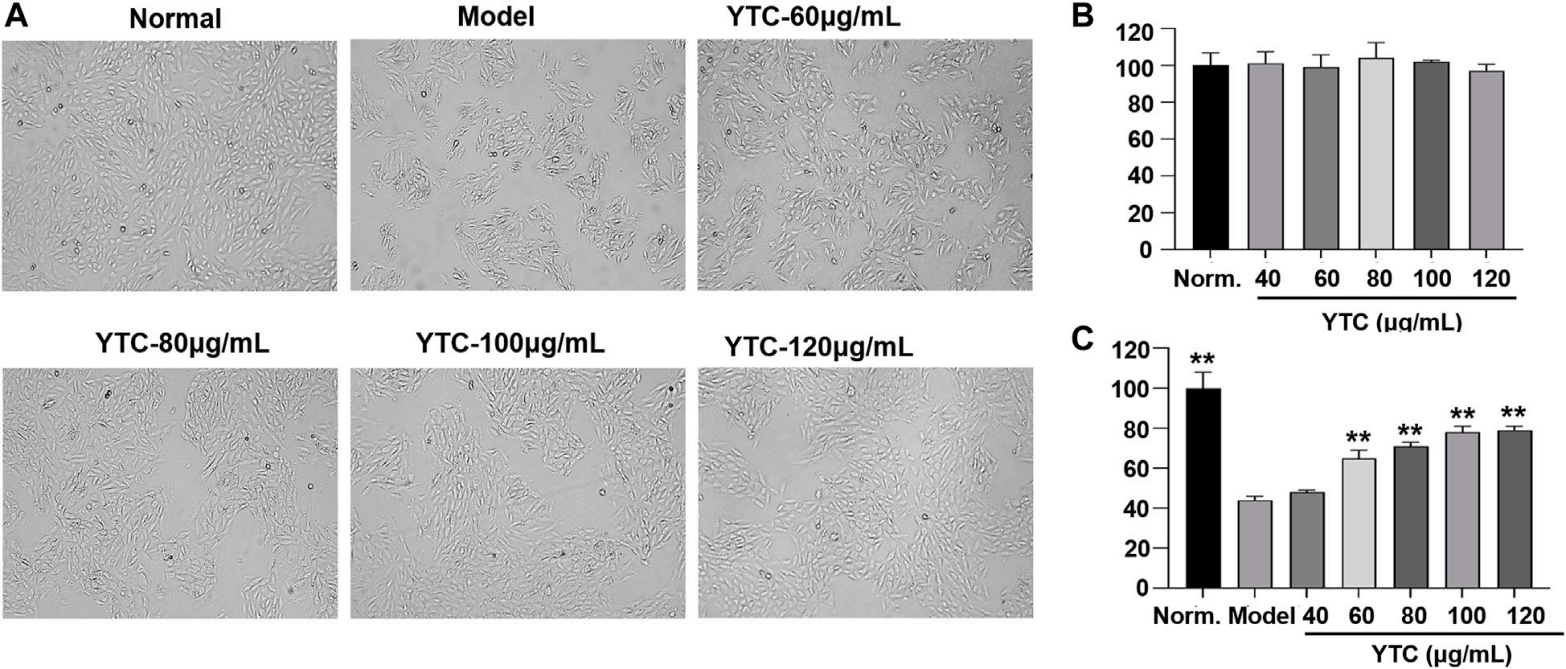
Protective effects of YTC on the cell viability of H_2_O_2_-stimulated H9c2 cells. **(A)** The represented cell morphology of H9c2 cells with different treatment. **(B)** Effects of YTC on cell viability of normal H9c2 cell. **(C)** Effects of YTC on cell viability of H_2_O_2_-stimulated H9c2 cells under different concentration. The values were represented as the mean ± SD (*n* = 3). ^∗∗^ *p* < 0.01 vs the model group.

The H9c2 cells were treated with different concentrations of YTC (0, 40, 60, 80, 100 and 120 μg/ml) for 24 h. As shown in Figure 5B, YTC at the different concentrations had no obvious damages on the viability of H9c2 cells compared with the normal group (*p* > 0.05). Whereas, after incubating with 200 μM H_2_O_2_ for 4 h, the viability of H9c2 cells decreased to nearly 40% compared with the normal group (*p* < 0.01) (Figures 5A,C). The viability of cells pretreated with YTC (60, 80, 100 and 120 μg/ml) for 24 h before stimulating by H_2_O_2_ concentration-dependently increased compared with the model group (*p* < 0.01) (Figures 5A,C).

#### Effects of YTC on Cell Apoptosis

**FIGURE 6:**
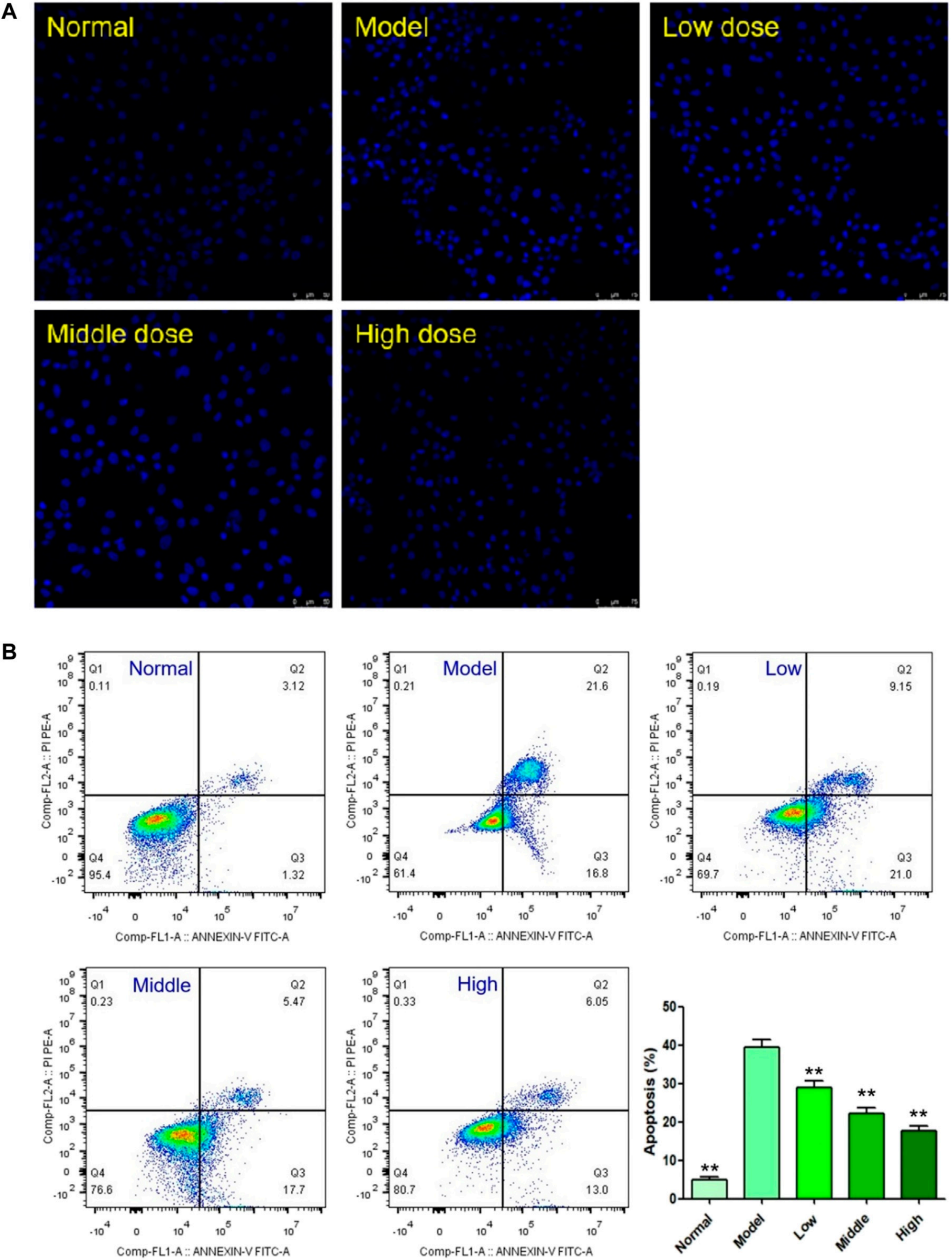
Effects of YTC on apoptosis in H_2_O_2_-stimulated H9c2 cells. **(A)** Apoptotic assay by DAPI staining. **(B)** Apoptotic assay by flow cytometry. The values were represented as the mean ± SD (*n* = 3). ^∗∗^ *p* < 0.01 vs the model group.

DAPI staining and flow cytometry were used to detect the effect of YTC on apoptosis in H9c2 cells. DAPI is a fluorescent dye which can penetrate cell membrane and is widely used in apoptosis assay. As shown in Figure 6A, under a fluorescence microscope, normal H9c2 cells were alive with round and intact nucleus and faint blue fluorescence. On the contrary, the nuclear of H_2_O_2_-stimulated cells shrank and showed obviously bright blue fluorescence, indicating characteristic apoptotic features. However, YTC treatment could significantly attenuate above morphological changes induced by H_2_O_2_.

The results of DAPI staining assay were also confirmed by flow cytometry assay. As shown in Figure 6B, 200 μM H_2_O_2_ triggered a high magnitude of apoptosis (38.40%), which was significantly higher than the rate in the normal group (4.44%, *p* < 0.01). However, the cell viability of YTC (60, 80 and 100 μg/ml) treatment group was significantly improved, compared with the model group (*p* < 0.01). At the YTC concentrations of 60, 80 and 100 μg/ml, there were 30.15, 23.17 and 19.05% apoptotic cells, respectively.

**FIGURE 7:**
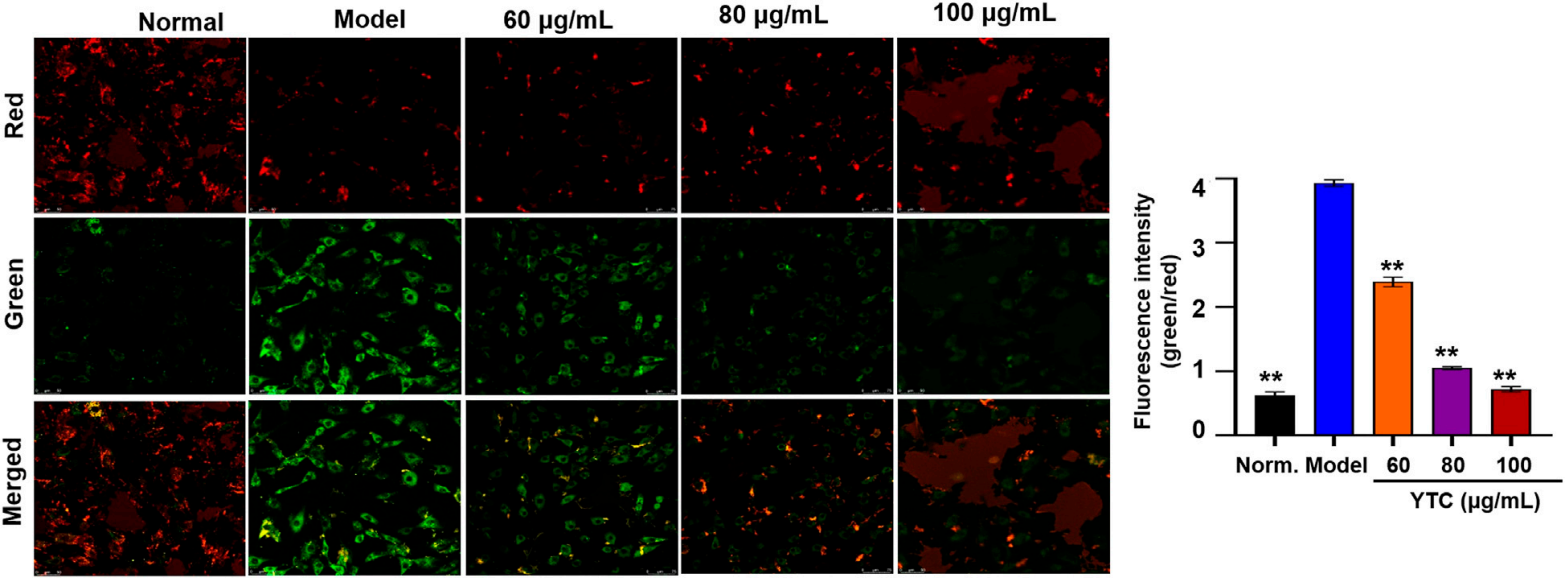
Effects of YTC on the ΔΨm in H9c2 cells (×40). Cells were pretreated with YTC (60, 80 and 100 μg/ml) for 24 h, and then incubated in the presence of H_2_O_2_ (200 μM) for 4 h ΔΨm was measured using a JC-1 assay kit and observed using a laser confocal microscopy under a 100× microscope. Data were expressed as the mean ± SD (n = 3). **p < 0.01 vs model group.

Mitochondrial membrane potential (MMP, ΔΨm), to a large extent, is considered an important index to judge whether the physiological function of mitochondria is normal. Generally, the decrease of MMP is considered to be one of the indicators of mitochondrial dysfunction and an early feature of apoptosis cells. In this experiment, the changes of MMP were measured by using a fluorescent probe called JC-1. When the physiological conditions are normal, JC-1 usually aggregates in the matrix of mitochondrial, forming a polymer emitting red fluorescence. When MMP is reduced, there showed green fluorescence, suggesting the polymer disintegrated. As shown in Figure 7, in the H_2_O_2_-stimulated cells, the red fluorescence decreased and the green fluorescence increased. In contrast, YTC-pretreatment improved the above changes, and the red fluorescence increased compared with the model group. These results suggested that YTC alleviated the apoptosis of H9c2 cells induced by H_2_O_2_.

#### Effects of Main Active Components on Cell Viability and Apoptosis

**FIGURE 8:**
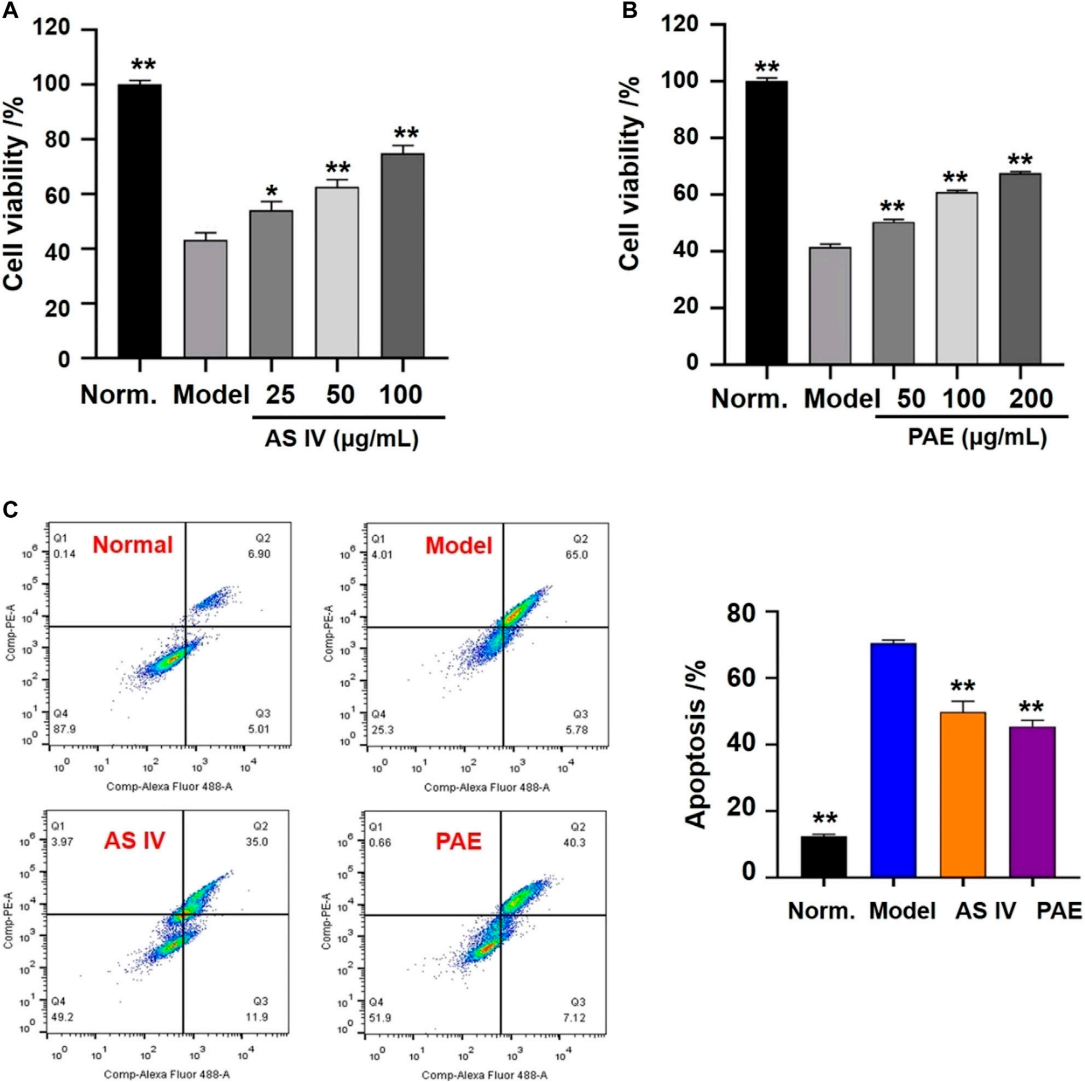
Effects of the main active components (AS IV and PAE) on cell viability and apoptosis in H_2_O_2_-stimulated H9c2 cells. **(A)** Effects of AS IV on cell viability of H2O2-stimulated H9c2 cells under different concentration (25, 50 and 100 μM). **(B)** Effects of PAE on cell viability of H2O2-stimulated H9c2 cells under different concentration (50, 100 and 200 μM). **(C)** Apoptotic assay by flow cytometry. The values were represented as the mean ± SD (*n* = 3). *p < 0.05, **p < 0.01 vs model group.

The effects of the two components (AS IV and PAE) on cell viability were shown in Figures 8A,B. After the treatment with H_2_O_2_, the viability of H9c2 cells in the model group decreased significantly compared with the normal group (*p* < 0.001), while pretreatment with different concentrations of AS IV (25, 50 and 100 μM) and PAE (50, 100 and 200 μM) improved the viability of H2O2 stimulated- H9c2 cells (*p* < 0.05). In addition, as shown in Figure 8C, after stimulating by H_2_O_2_, the apoptosis rate of H9c2 cells in the model group was significantly higher than that of normal group (*p* < 0.001), while the apoptosis rate was decreased after pretreatment with the two main active components respectively (*p* < 0.001).

#### Effects of YTC on Intracellular ROS Generation

**FIGURE 9:**
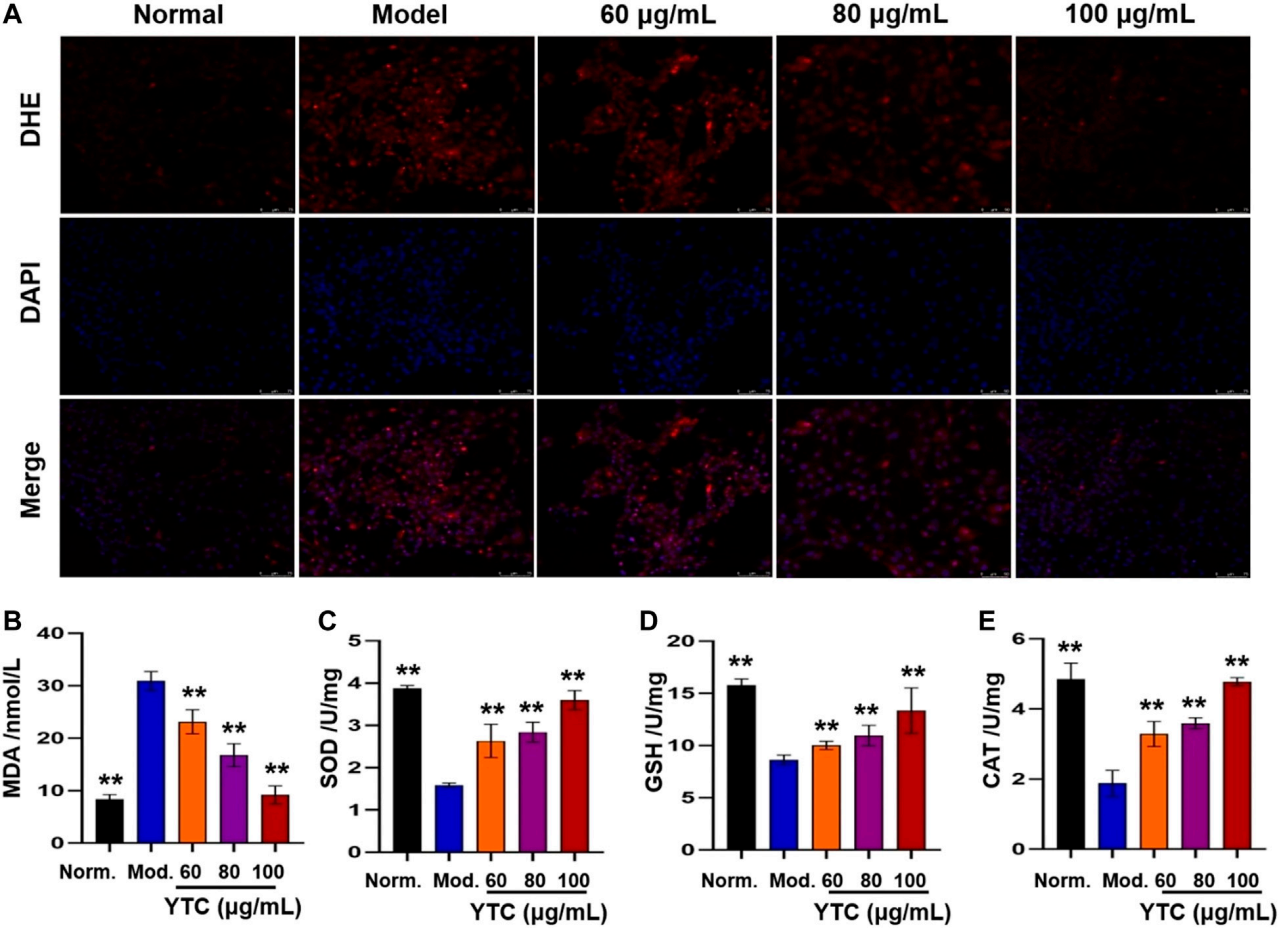
Effects of YTC on ROS levels **(A)** and antioxidant enzyme activities **(B-E)** in H_2_O_2_-induced H9c2 cells. H9c2 cells were treated with YTC (60, 80 and 100 μg/ml) for 24 h, and then incubated in the presence of H_2_O_2_ (200 μM) for 4 h **(A)**The intracellular ROS levels were observed by laser confocal microscopy. **(B-E)**The levels of MDA and activities of SOD, GSH-Px, and CAT were determined by commercial assay kits. The values were represented as the mean ± SD (*n* = 3). ^**^ *p* < 0.01 vs the model group.

Excessive ROS will be produced after cells stimulating by H_2_O_2_, which is an important cause of cell apoptosis (Ogura and Shimosawa, 2014). Therefore, we used fluorescence probe DHE to detect the level of ROS. As shown in Figure 9A, compared with the normal group, the model group showed obvious red fluorescence, suggesting intracellular superoxide anion levels increased. Interestingly, the above phenomena could be alleviated by pretreatment with YTC (60, 80 and 100 μg/ml).

#### Effect of YTC on Antioxidant Enzyme

Oxidative stress injury is one of the important mechanisms of MI injury (Xie et al., 2021). In order to determine whether YTC affects biochemical enzymes related to oxidative stress, we measured the levels of intracellular lipid peroxidation products (MDA) and antioxidant enzymes (SOD, GSH-Px and CAT). As shown in Figure 9B, the content of MDA in H_2_O_2_-stimulated H9c2 cells was significantly increased compared with the normal group (*p* < 0.01). Whereas, YTC (60, 80 and 100 μg/ml) could alleviate this trend, compared to the model group (*p* < 0.01). In addition, the activities of SOD, GSH-Px and CAT in H_2_O_2_-treated cells were decreased compared with that in the normal group (*p* < 0.01), while the activities of the three enzymes were increased after YTC pretreatment (*p* < 0.01). The results indicated that YTC might have antioxidant capacity to protecting H9c2 cells from H_2_O_2_ damage.

### Molecular Mechanism of the Protective Effects of YTC on H_2_O_2_-Treated H9c2 Cells

**FIGURE 10:**
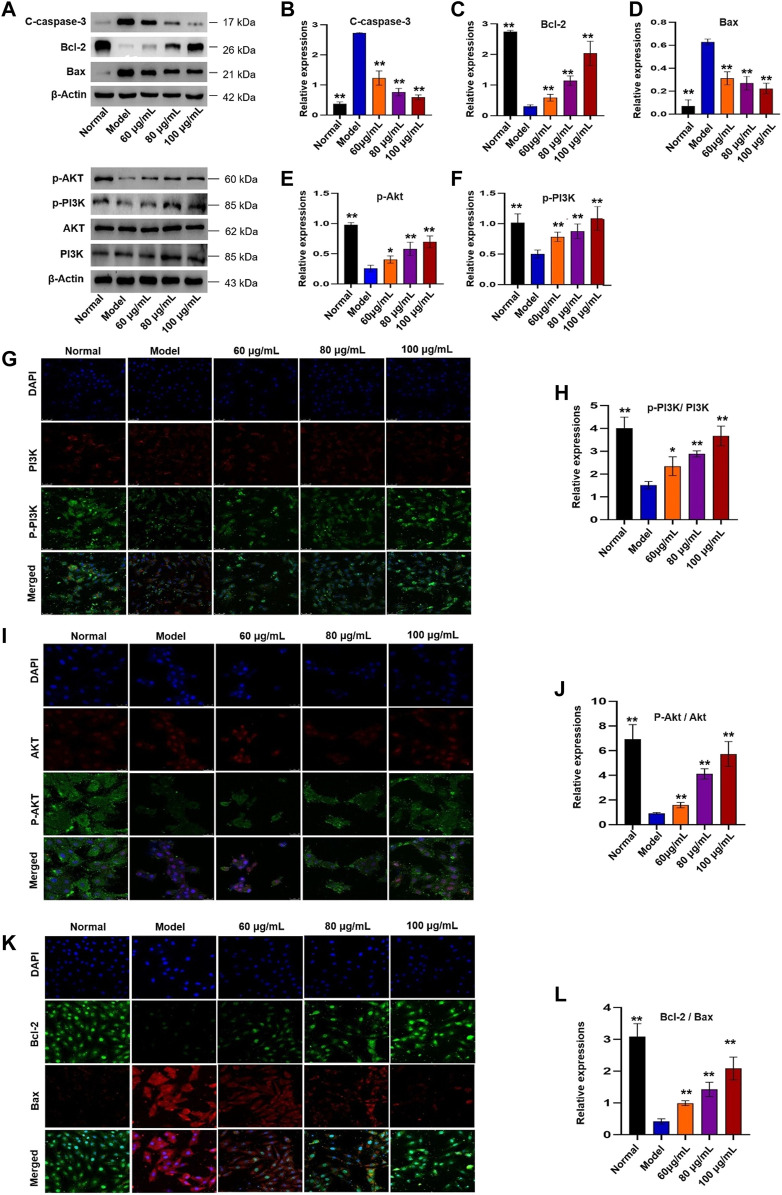
Effects of YTC on PI3K/Akt signaling pathway in H_2_O_2_-induced H9c2 cells. **(A)** WB images of C-caspase-3, Bcl-2, Bax, p-Akt, p-PI3K, AKT and PI3K protein expression. **(B)** Analysis of C-caspase-3. **(C)** Analysis of Bcl-2. **(D)** Analysis of Bax. **(E)** Analysis of p-Akt. **(F)** Analysis of p-PI3K. **(G)** IF images of p-PI3K and PI3K. **(H)** Analysis of the p-PI3K/PI3K ratio. **(I)** IF images of p-Akt and Akt. **(J)** Analysis of the p-Akt/Akt ratio. **(K)** IF images of Bcl-2 and Bax. **(L)** Analysis of the Bcl-2/Bax ratio. Data were expressed as mean ± SD (n = 3), **p < 0.05 and **p < 0:01 vs model.

The KEGG analysis indicated that apoptosis and PI3K/Akt signaling pathway played an important role in YTC against MI. Therefore, western blot and immunofluorescence were utilized to evaluate the results. In Figure 10, the expressions of Bcl-2, Bcl-2/Bax, p-PI3K and p-Akt in the H_2_O_2_-stimulated model group were decreased, while the expressions of Bax and C-caspase-3 were increased, compared with the normal group (*p* < 0.01). However, 60, 80 and 100 μg/ml YTC could reverse these changes (*p* < 0.01). The expression of PI3K and Akt has no significant change in each group.

**FIGURE 11:**
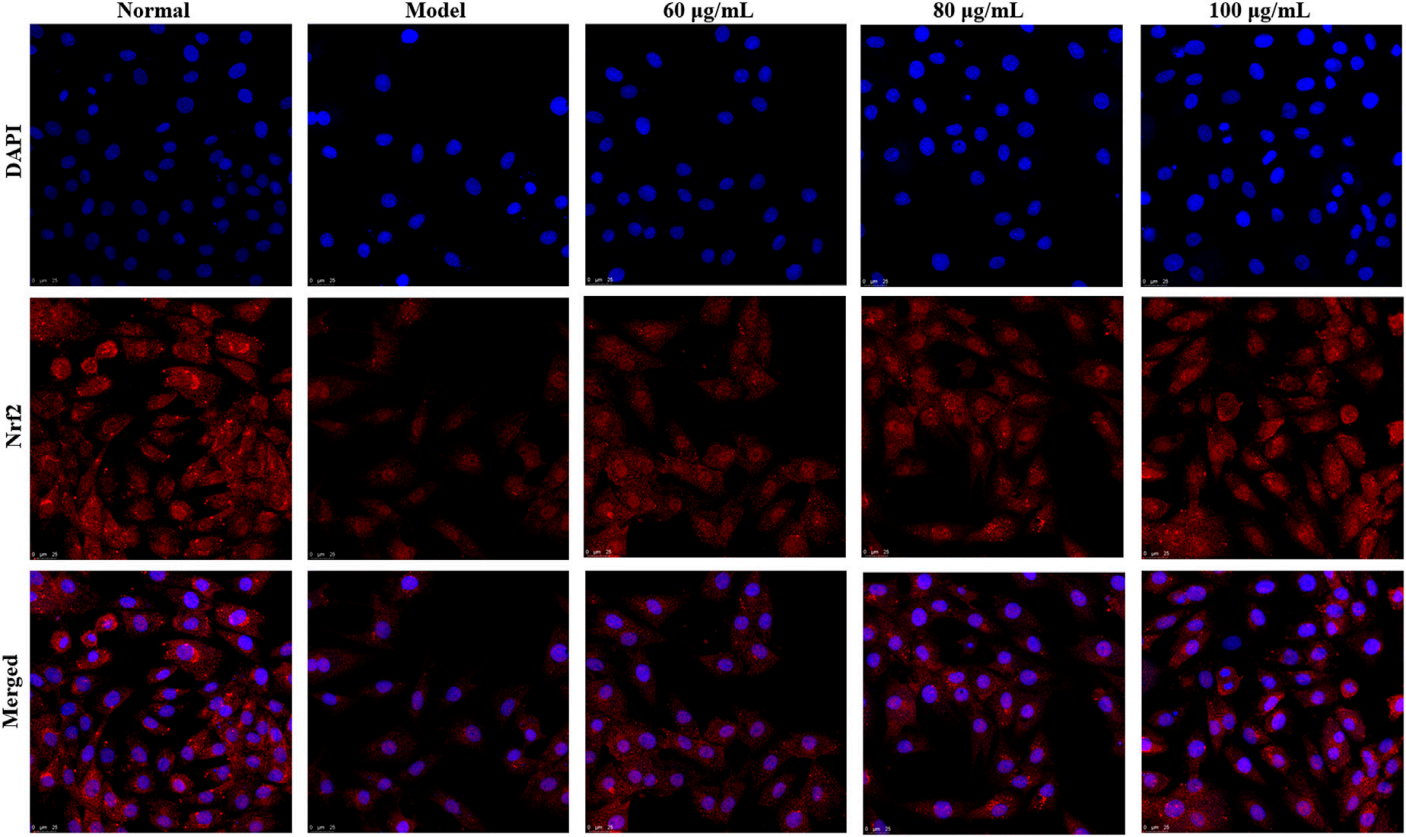
Effects of YTC on nuclear transcription of Nrf2 in H_2_O_2_-induced H9c2 cells. H9c2 cells were treated with YTC (60, 80 and 100 μg/ml) for 24 h, subsequently subjected to H_2_O_2_ (200 μM) for 4 h. The nuclear transcription of Nrf2 was determined by laser confocal microscopy.

In addition, Nrf2 plays an important role in the regulation of redox homeostasis, so we further examined the effect of YTC on Nrf2 nucleation in H_2_O_2_-induced H9c2 cells. The results of immunofluorescence staining (Figure 11) showed that after pre-treatment with YTC, Nrf2 was mainly located in the nucleus compared with the model group, indicating that YTC might activate Nrf2 to transfer to the nucleus. The above results suggested that YTC might protect H_2_O_2_-induced H9c2 cells form oxidative stress and apoptosis by the PI3K/Akt/Nrf2 signalling pathway.

## Discussion

Currently, increasing reports have indicated that natural medicines, particularly the TCMs, are promising resources for finding useful agents for treating various diseases (Mozaffarian and Wu, 2018; Wang et al., 2019). One of the characteristics of TCM is that different components and targets play a role in different pathways, which makes it a challenge to study the molecular mechanisms of TCM. Network analysis has the characteristics of integrity and systematicness, which builds a bridge for different drugs to study interactions between them and is very suitable to research TCM. In this study, 162 potential compounds were collected from online databases. Then the 162 compounds were uploaded to TCMSP and SwissTarget online databases, and 89 targets were predicted, while disease targets were collected from OMIM and Genecards database. According to the DMTD diagram and previous reports, AS IV and PAE were considered might be the key components of YTC against MI. It has been reported that AS IV could protect heart from MI and reperfusion injury through a variety of ways, including regulating autophagy, oxidative stress and energy metabolisms (Tu et al., 2013; Jiang et al., 2019; Huang et al., 2021). In addition, a study suggested that PAE had protective effect on myocardial ischemia-reperfusion by inhibiting apoptosis and reducing oxidative stress (Wu et al., 2020). *In vitro*, the results of CCK-8 assay and flow cytometry showed that the above two components significantly improved the decrease of cell viability and alleviated apoptosis in the H_2_O_2_-induced H9c2 cells.

The results of GO and KEGG analysis showed that the potential molecular mechanisms and signal pathways of YTC against MI were strongly related to apoptosis signal pathway. Similarly, the PPI analysis showed that the core target proteins of active ingredients in YTC were related to apoptosis, such as CASP3, CASP8, CASP9 and BCL2. Accumulated studies have also indicated that myocardial apoptosis was important in MI (Garg et al., 2003; Wang et al., 2013; Li et al., 2018). The results of GO analysis showed that oxidative stress may be related to mechanisms of YTC to against MI. Oxidative stress leads to excessive production of ROS, and then damages myocardial cells, which is an inevitable process in the pathogenesis of cardiovascular disease (Heistad et al., 2009; Zhang et al., 2018). Moreover, PI3K/Akt signaling pathway play the role of anti-oxidant stress by regulating Nrf2 (Li et al., 2016). Briefly, activated Akt leads to Nrf2 release from the Keap1-Nrf2 complex to the nucleus, and then Nrf2 reacts with the antioxidant response element sequences to protect the cell against oxidative stress (Li et al., 2018). Interestingly, KEGG results showed that PI3K/Akt pathway, an apoptosis-related signaling pathway, had a high anti-MI correlation in YTC, suggesting YTC may play an anti-MI role via alleviating oxidative stress and inhibiting apoptosis of cardiomyocyte via the PI3K/Akt pathway. In order to verify this hypothesis, *in vitro* studies were performed to verify the predicted results by network analysis.

In this study, we used H_2_O_2_ stimulated H9c2 cells to establish an oxidative stress injury model. The viability of H9c2 cells decreased significantly after incubation with H_2_O_2_; however, the decrease of cell viability induced by H_2_O_2_ reversed by pre-treatment with YTC. In addition, H_2_O_2_ stimulation significantly increased the accumulation of ROS in H9c2 cells, while pretreatment with YTC reduced the excessive accumulation of ROS. MDA is a biomarker of oxidative stress (Valko et al., 2007). SOD, CAT and GSH-Px are important ROS scavenging enzyme, which could maintain the redox balance in body (Chance et al., 1979). According to our results, in H_2_O_2_-timulated H9c2 cells, the content of MDA increased, while the activities of SOD, GSH-Px and CAT decreased. On the contrary, pretreatment with YTC reversed the above changes. In addition, after pretreatment with YTC, significant apoptosis of H9c2 cells induced by H_2_O_2_ alleviated. These results suggested that YTC may protect H9c2 cells from ROS-induced apoptosis.

**FIGURE 12:**
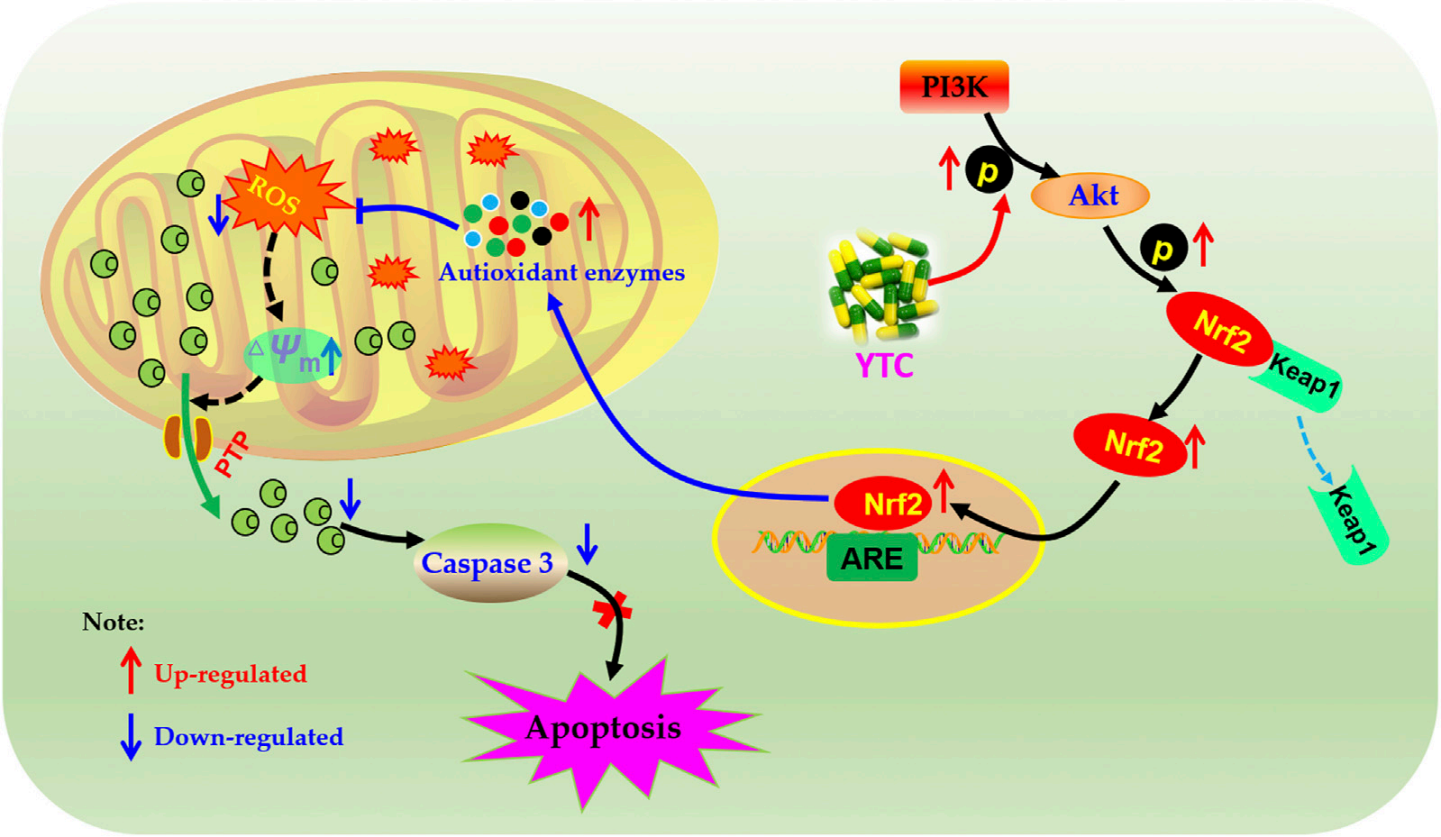
Molecular mechanism of the protective effect of YTC. YTC possesses protective potentials on H_2_O_2_-stimulated H9c2 cells through suppression of oxidative stress-induced apoptosis via regulation of the PI3K/Akt/Nrf2 signal pathway.

Accumulating studies have suggested that the PI3K/Akt signalling pathway is important in regulating ROS expression and cellular oxidative stress pathways (Le Belle et al., 2011; Tsai et al., 2013; Lei et al., 2019). PI3K is an important signal transduction molecule in the growth factor superfamily, and Akt is a serine/threonine kinase, which is a key mediator of PI3K-mediated signal transduction (Lei et al., 2019). The result of PI3K phosphorylation is phosphorylation of Akt, which will affect the expression of Bcl-2 and Bax proteins. Bcl-2 and Bax are a group of proteins closely related to apoptosis, and the anti-apoptotic protein Bcl-2 can inhibit the proapoptotic effect of Bax (Kønig et al., 2019). In the PI3K/Akt signalling, the expression of Bcl-2 increased and the expression of Bax decreased by activated p-Akt. In addition, Nrf2 was reported to be an essential protein for the production of antioxidant enzymes (Zhang et al., 2021b). Normally, Nrf2 is localized in the cytoplasm with keap 1. However, under conditions of oxidative stress, the structure of Keap1 is changed, resulting in the dissociation of Nrf2 from Keap1 into the nucleus. In the nucleus, Nrf2 combines with ARE to activate transcription to generate various antioxidant enzymes in the cytoplasm, including SOD, HO-1, CAT and GSH (Wen et al., 2018). In addition, PI3K/Akt is the main signaling pathway to activate Nrf2 (Zhang et al., 2021b). Our results showed that pretreatment with YTC promoted Nrf2 nuclear translocation and up-regulated the phosphorylation levels of PI3K and Akt, suggesting that the scavenging effect of YTC on ROS was closely related to the regulation of PI3K/Akt/Nrf2 signaling pathway in H_2_O_2_ stimulated-H9c2 cells (Figure 12).

## Conclusion

In this study, network analysis and *in vitro* experiments were adopted to determine the active components and molecular mechanisms of YTC against MI. 162 compounds were obtained and the potential targets of these compounds were screened from online databases, and the intersection targets with the disease targets obtained from online databases were taken as the potential targets of YTC against MI. According to the analysis of PPI network and DMTD diagram, as well as GO and KEGG enrichment analysis, it is concluded that AS IV and PAE might be the most important component in YTC; YTC might play an anti-MI role by inhibiting apoptosis induced by oxidative stress via the PI3K/Akt/Nrf2 signal pathway. The results of *in vitro* experiments also verified the results of network analysis prediction. In conclusion, this study clarified the anti-MI mechanisms of YTC, and provided references for the research and promotion of Chinese traditional patent medicines.

## Data Availability

The original contributions presented in the study are included in the article/Supplementary Material, further inquiries can be directed to the corresponding authors.
