# Supplementary material for: An Integrated Approach Based on Network Analysis Combined With Experimental Verification Reveals PI3K/Akt/Nrf2 Signaling Is an Important Way for the Anti-Myocardial Ischemia Activity of Yi-Qi-Tong-Luo Capsule
Source: Front Pharmacol. 2022 Feb 16;13:794528. doi: 10.3389/fphar.2022.794528 (PMC8889021; doi:10.3389/fphar.2022.794528)
Supplement: Supplementary file 3 [file Table1.docx]

**Supplementary Table S1 |** Potential Active Compounds in YTC.

| **No.** | **TCMSP ID** | **Compound** |
| --- | --- | --- |
|  | MOL001689 | Acacetin |
|  | MOL000471 | Aloe-emodin |
|  | MOL002881 | Diosmetin |
|  | MOL005190 | Eriodictyol |
|  | MOL011616 | Fortunellin |
|  | MOL005573 | Genkwanin |
|  | MOL001790 | Linarin |
|  | MOL007330 | L-Menthol |
|  | MOL007330 | L-Menthone |
|  | MOL004328 | Naringenin |
|  | MOL006990 | (1S,2S,4R)-trans-2-hydroxy-1,8-cineole-β-D-glucopyranoside |
|  | MOL006992 | (2R,3R)-4-methoxyl-distylin |
|  | MOL006994 | 1-O-β-D-glucopyranosyl-8-o-benzoylpaeonisuffrone |
|  | MOL006996 | 1-O-β-D-glucopyranosylpaeonisuffrone |
|  | MOL007008 | 4-ethyl-paeoniflorin |
|  | MOL007012 | 4-O-methyl-paeoniflorin |
|  | MOL007014 | 8-debenzoylpaeonidanin |
|  | MOL007018 | 9-ethyl-neo-paeoniaflorin A |
|  | MOL007005 | Albiflorin |
|  | MOL002714 | Baicalein |
|  | MOL002776 | Baicalin |
|  | MOL007003 | Benzoyl paeoniflorin |
|  | MOL005043 | Campest-5-en-3β-ol |
|  | MOL000492 | Catechin |
|  | MOL001002 | Ellagic acid |
|  | MOL002883 | Ethyl oleate |
|  | MOL007022 | Evofolin B |
|  | MOL007025 | Isobenzoylpaeoniflorin |
|  | MOL001921 | Lactiflorin |
|  | MOL001918 | Paeoniflorgenone |
|  | MOL001925 | Paeoniflorin |
|  | MOL006999 | Stigmast-7-en-3-ol |
|  | MOL002719 | 6-Hydroxynaringenin |
|  | MOL002757 | 7,8-dimethyl-1H-pyrimido[5,6-g]quinoxaline-2,4-dione |
|  | MOL002189 | Butylphthalide |
|  | MOL002717 | Carthamone |
|  | MOL002127 | Cnidilide |
|  | MOL002680 | Flavoxanthin |
|  | MOL002733 | Hydroxysafflor Yellow A |
|  | MOL002695 | Lignan |
|  | N/A | Ligusticoside B |
|  | MOL011782 | Ligustilide |
|  | MOL002698 | Lupeol-palmitate |
|  | MOL001494 | Mandenol |
|  | MOL002135 | Myricanone |
|  | MOL002706 | Phytoene |
|  | MOL002707 | phytofluene |
|  | MOL001771 | Poriferast-5-en-3beta-ol |
|  | MOL002710 | Pyrethrin II |
|  | MOL002721 | Quercetagetin |
|  | MOL002208 | Senkyunolide A |
|  | MOL011784 | Senkyunolide B |
|  | MOL002143 | Senkyunolide C |
|  | MOL002144 | Senkyunolide D |
|  | MOL002146 | Senkyunolide F |
|  | MOL002151 | Senkyunone |
|  | MOL002202 | Tetramethylpyrazine |
|  | MOL002157 | Wallichilide |
|  | MOL001945 | 5-Methoxypsoralen |
|  | MOL011782 | Ligustilide |
|  | MOL002143 | Senkyunolide I |
|  | MOL008411 | 11-Hydroxyrankinidine |
|  | MOL007059 | 3-β-Hydroxymethyllenetanshiquinone |
|  | MOL008391 | 5-α-Stigmastan-3,6-dione |
|  | MOL008393 | 7-(β-Xylosyl)cephalomannine_qt |
|  | MOL003896 | 7-Methoxy-2-methyl isoflavone |
|  | MOL004492 | Chrysanthemaxanthin |
|  | MOL008397 | Daturilin |
|  | MOL002879 | Diop |
|  | MOL005321 | Frutinone A |
|  | MOL008400 | Glycitein |
|  | MOL000006 | Luteolin |
|  | MOL007514 | Methyl icosa-11,14-dienoate |
|  | MOL002140 | Perlolyrine |
|  | MOL001006 | Poriferasta-7,22 E-dien-3β-ol |
|  | MOL008406 | Spinoside A |
|  | MOL006774 | Stigmast-7-enol |
|  | MOL008379 | Tangshenoside I |
|  | MOL006554 | Taraxerol |
|  | MOL005320 | Arachidonic acid |
|  | MOL005448 | Leucine |
|  | MOL000421 | Nicotinic acid |
|  | MOL000438 | (3R)-3-(2-hydroxy-3,4-dimethoxy-phenyl) chroman-7-ol |
|  | MOL000442 | 1,7-Dihydroxy-3,9-dimethoxy pterocarpene |
|  | MOL000374 | 5'-hydroxy-iso-muronulatol-2',5'-di-O-glucoside |
|  | MOL000378 | 7-O-methyl-iso-mucronulatol |
|  | MOL000379 | 9,10-di-methoxy-pterocarpan-3-O-β-D-glucoside |
|  | MOL000407 | Astragaloside I |
|  | MOL000403 | Astragaloside II |
|  | MOL000405 | Astragaloside III |
|  | MOL000407 | Astragaloside IV |
|  | MOL000417 | Calycosin |
|  | MOL000392 | Formononetin |
|  | MOL000296 | Hederagenin |
|  | N/A | Isoastragaloside I |
|  | N/A | Isoastragaloside I I |
|  | MOL000398 | Isoflavanone |
|  | MOL000439 | Iso-mucronulatol-7,2'-di-O-glucosiole |
|  | MOL000354 | Isorhamnetin |
|  | MOL000239 | Jaranol |
|  | MOL000211 | Mairin |
|  | MOL001040 | (2R)-5,7-dihydroxy-2-(4-hydroxyphenyl)chroman-4-one |
|  | MOL000228 | (2R)-7-hydroxy-5-methoxy-2-phenylchroman-4-one |
|  | MOL002940 | (3R)-3-(2,3-dihydroxy-4-methoxyphenyl)-7-hydroxychroman-4-one |
|  | MOL002941 | (3R)-3-(2,3-dihydroxy-4-methoxyphenyl)chroman-7,8-diol |
|  | MOL002938 | (3R)-4'-Methoxy-2',3,7-trihydroxyisoflavanone |
|  | MOL002939 | (3R)-5'-Methoxyvestitol |
|  | MOL002950 | (3R)-7,2',3'-trihydroxy-4-methoxyisoflavan |
|  | MOL002982 | (3R,4R)-3',7-dihydroxy-2',4'-dimethoxy-4-[(2S)-4',5,7-trihydroxyflavanone-6-yl]isoflavan |
|  | MOL002962 | (3S)-7-hydroxy-3-(2,3,4-trimethoxyphenyl)chroman-4-one |
|  | MOL002990 | (6aR,11aR)-3,9,10-trimethoxy-6a,11a-dihydro-6H-benzofurano[3,2-c]chromen-4-ol |
|  | MOL002991 | (6aR,11aR)-3,9-dimethoxy-6a,11a-dihydro-6H-benzofurano[3,2-c]chromene-4,10-diol |
|  | MOL000380 | (6aR,11aR)-9,10-dimethoxy-6a,11a-dihydro-6H-benzofurano[3,2-c]chromen-3-ol |
|  | MOL002997 | 3-(2-hydroxy-3,4-dimethoxyphenyl)-2H-chromen-7-ol |
|  | MOL002958 | 3'-Hydroxymelanettin |
|  | MOL002959 | 3'-Methoxydaidzein |
|  | MOL002963 | 4',5',7-trimethyl-3-methoxyflavone |
|  | MOL002989 | 4-Hydroxyhomopterocarpin |
|  | MOL002967 | 7-hydroxy-4'-methoxy-2',5'-dioxo-4-[(3R)-2',7-dihydroxy-4'-methoxyisoflavan-5'-yl]isoflavane |
|  | MOL002957 | 9-O-Methylcoumestrol |
|  | MOL002973 | Bowdichione |
|  | MOL002975 | Butin |
|  | MOL002966 | Dalbergin |
|  | MOL001792 | DFV |
|  | MOL002981 | Duartin |
|  | MOL002985 | Isoduartin |
|  | MOL002565 | Medicarpin |
|  | MOL002996 | Odoricarpin |
|  | MOL002999 | Sativanone |
|  | MOL003000 | Stevein |
|  | MOL002961 | Vestitol |
|  | MOL003001 | Vestitone |
|  | MOL003002 | Violanone |
|  | MOL003003 | Xenognosin B |
|  | MOL001368 | 3-O-p-coumaroylquinic acid |
|  | MOL001368 | Amygdalin |
|  | MOL000493 | Campesterol |
|  | MOL001371 | Populoside |
|  | MOL000483 | (Z)-3-(4-hydroxy-3-methoxy-phenyl)-N-[2-(4-hydroxyphenyl)ethyl]acrylamide |
|  | MOL000332 | Coumaroyltyramine |
|  | MOL002341 | Hesperetin |
|  | MOL007640 | Macrostemonoside E |
|  | MOL007651 | Prostaglandin B1 |
|  | MOL001973 | Sitosteryl acetate |
|  | MOL002733 | Hydroxysafflor yellow A |
|  | MOL004718 | α-spinasterol |
