# Supplementary material for: An Integrated Approach Based on Network Analysis Combined With Experimental Verification Reveals PI3K/Akt/Nrf2 Signaling Is an Important Way for the Anti-Myocardial Ischemia Activity of Yi-Qi-Tong-Luo Capsule
Source: Front Pharmacol. 2022 Feb 16;13:794528. doi: 10.3389/fphar.2022.794528 (PMC8889021; doi:10.3389/fphar.2022.794528)
Supplement: Supplementary file 4 [file DataSheet1.docx]

***HPLC-Q Exactive Orbitrap-MS Method for Component Analysis***

The main chemical components of YTC were determined by HPLC-Q-Exactive-Orbitrap-MS analysis. HPLC analysis was carried out applying an a Thermo Scientific Ultimate 3000 RSLCnano system (Thermo Fisher Scientific, USA), and a CAPCELL PAK MG II S5 C18 (250×4.6 mm, i.d.; 5 μm, Shiseido, Tokyo, Japan) was applied for sample separation at 35°C. The gradient elution system consisted Acetonitrile (A) and 0.1% formic acid-water (B) with a flow rate of 0.2 mL/min, and the sample input volume was set at 10.0 μL. The gradient program was set as: 0–5 min, 5% A; 5–15 min, 5%-15% A; 15–25 min, 15%-30% A; 25–30 min, 30–55% A; 30–40 min, 55%-75% A; 40–45 min, 75–85% A; 45–50 min, 85% A. Mass analysis was performed on a Q-Exactive (Thermo Scientific, San Jose, CA, USA). Nitrogen was applied as auxiliary gas and sheath gas with the flow rate of 10 L/min. The mass determination was carried out based on positive and negative scanning mode with the m/z ranging from 100 to 1200.

***Result***

The HPLC-Q Exactive Orbitrap-MS assay of the YTC extracts was carried out and the results showed the main constituents in YTC are ligustilide, formononetin, Naringenin and isoliquiritigenin, *etc* (**Supplementary Figure S1**).


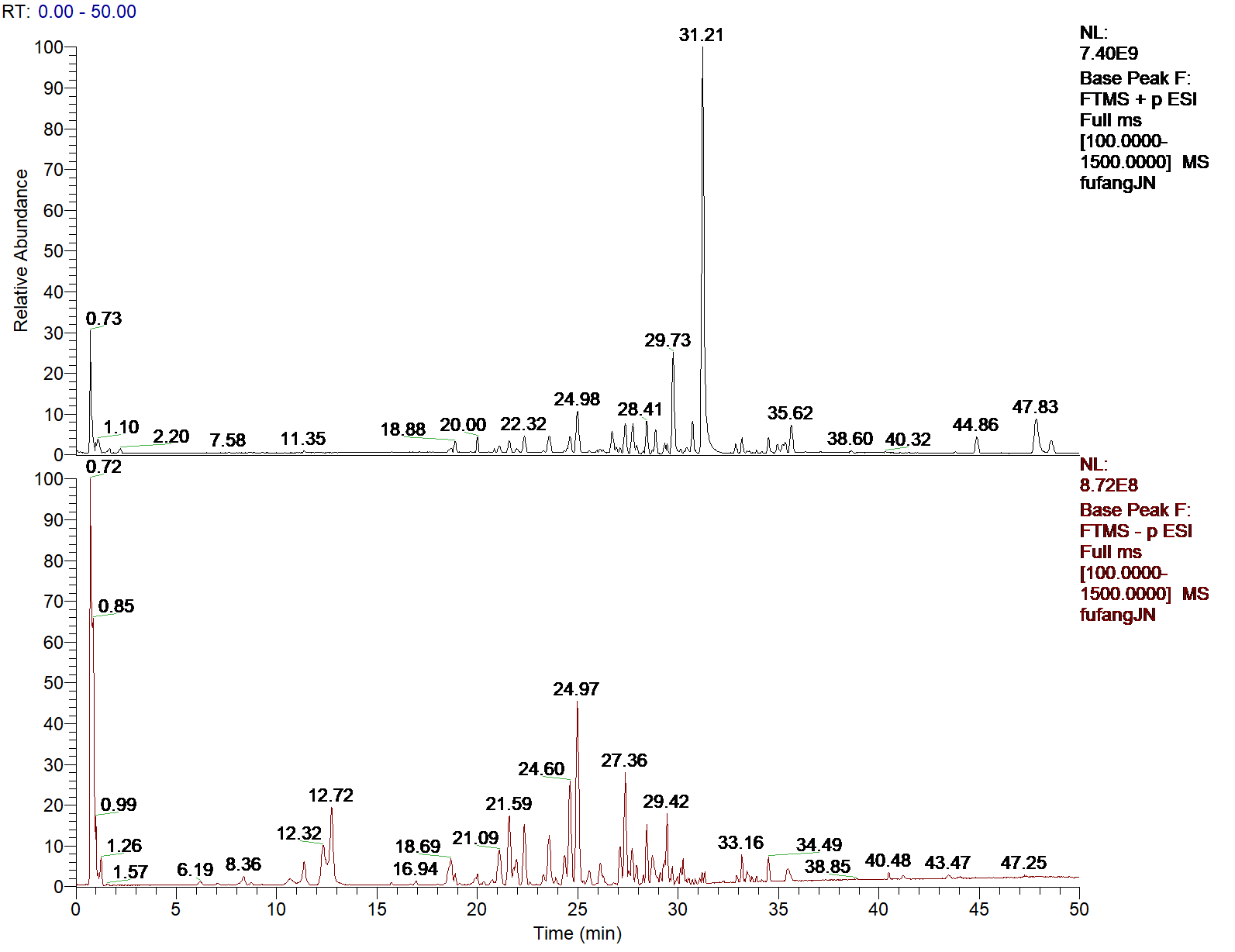


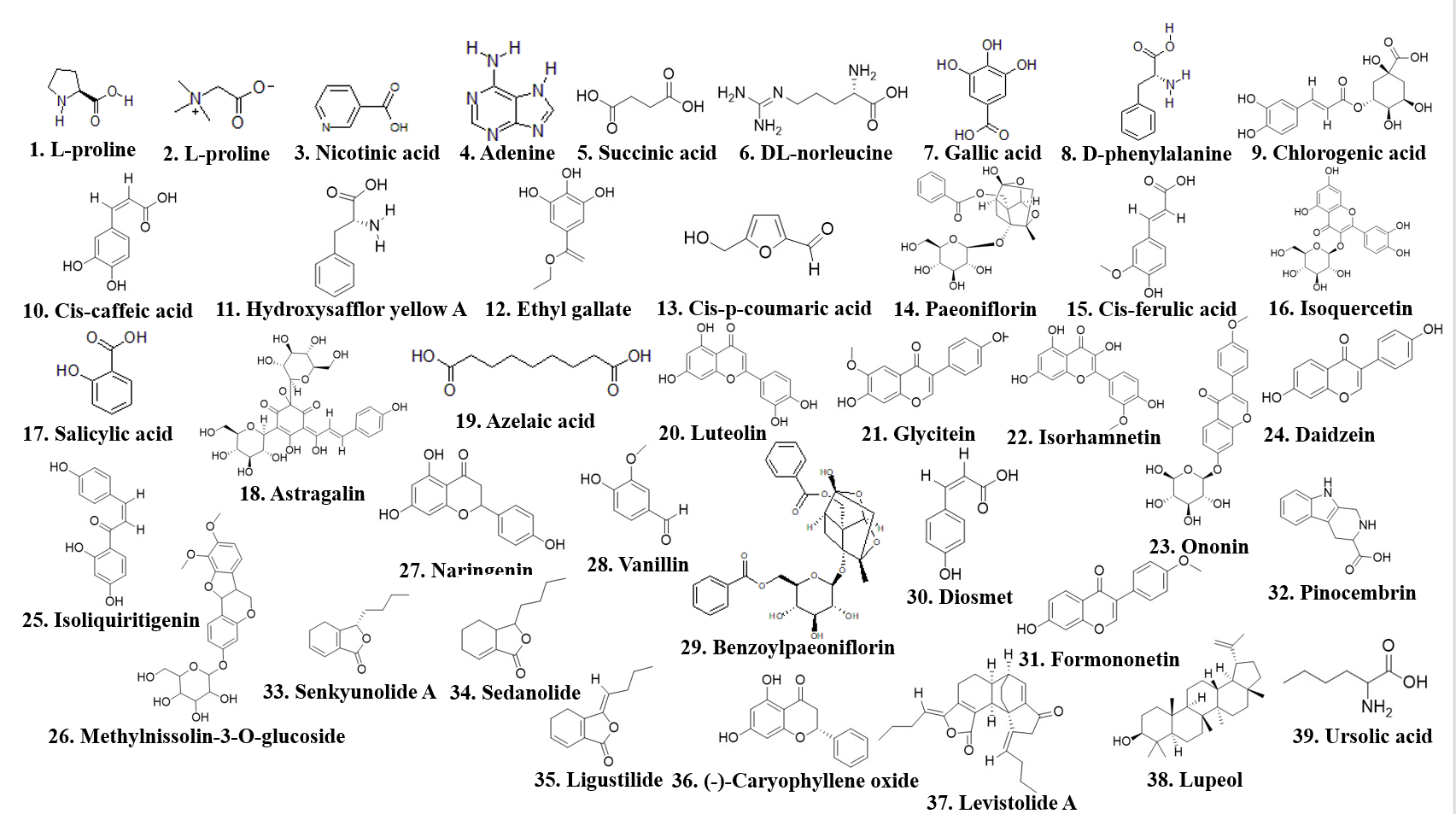


**Supplementary Figure S1 |** Result of the HPLC-Q-Exactive-Orbitrap-MS analysis of YTC.

L-proline (1), betaine (2), nicotinic acid (3), adenine (4), succinic acid (5), DL-norleucine (6), gallic acid (7), D-phenylalanine (8), chlorogenic acid (9), cis-caffeic acid (10), hydroxysafflor yellow A (11), ethyl gallate (12), cis-p-coumaric acid (13), paeoniflorin (14), cis-ferulic acid (15), isoquercetin (16), salicylic acid (17), astragalin (18), azelaic acid (19), luteolin (20), glycitein (21), isorhamnetin (22), ononin (23), daidzein (24), isoliquiritigenin (25), methylnissolin-3-O-glucoside (26), naringenin (27), vanillin (28), benzoylpaeoniflorin (29), diosmetin (30), formononetin (31), pinocembrin (32), senkyunolide A (33), sedanolide (34), ligustilide (35), (-)-caryophyllene oxide (36), levistolide A (37), lupeol (38), ursolic acid (39).
